# Gender authorship of articles in pediatric ophthalmology and strabismus between 2002 and 2018

**DOI:** 10.1038/s41433-021-01397-1

**Published:** 2021-02-19

**Authors:** Baharav Einav, Shemesh Rachel, Magnezi Ofir, Mezer Eedy, Wygnanski-Jaffe Tamara

**Affiliations:** 1 Sackler Faculty of Medicine, Tel Aviv University, Tel Aviv, Israel; 2 Department of Ophthalmology, Sheba Medical Center, Tel Hashomer, Israel; 3 Saint George’s, University of London Medical School, London, UK; 4 Department of Ophthalmology, Rambam Health Care Campus, Haifa, Israel; 5 Bruce and Ruth Rappaport Faculty of Medicine, Technion—Israel Institute of Technology, Haifa, Israel

**Keywords:** Outcomes research, Events

## To the Editor:

This study evaluated trends over time of women representation in authorship positions of academic publications in pediatric ophthalmology and strabismus (POS) to compare it to the increase in female authors reported in studies the field of ophthalmology [1, 2].

We selected the 10 highest ranked journals in ophthalmology categories (Table 1) according to their impact factor (IF) and quartile (Q) ranking, listed by the Journal Citation Reports (2018). We collected all articles published on POS from January 1, 2002 through December 31, 2018.

**Table 1:** Ranking of 10 preselected journals according to their impact factor (IF) and their quartile (Q) ranking in ophthalmology and pediatric categories, as listed by the Journal Citation Reports (2018).

Quartile ophthalmology 2018	Impact Factor 2018	Rank Order	Journal Name
Q1	7.732	1	*Ophthalmology*
Q1	6.167	2	*JAMA ophthalmology*
Q1	4.483	3	*American Journal of Ophthalmology*
Q1	3.615	4	*British Journal of Ophthalmology*
Q1	3.153	5	*Acta Ophthalmologica*
Q2	2.366	6	*Eye*
Q2	2.25	7	*Graefe’s archive for clinical and experimental ophthalmology*
Q4	1.056	8	*Journal of American association for pediatric ophthalmology and strabismus*
Q4	1.054	9	*Journal of pediatric ophthalmology and strabismus*
-	-	10	*Strabismus*

We utilized the Baby Name Guesser software (available at: http://www.gpeters.com/names/baby-names.php). SPSS software (IBM SPSS Statistics for Windows, ver. 24, IBM corp., Armonk, NY, USA, 2016) was used. All tests were two sided. *P* < 0.05 was considered statistically significant. We used the Generalized Estimating Equations (GEE) model for multivariate analysis of trends in various categories of variables significantly associated with author gender.

We analysed 30,724 authors from 6617 published articles. Final analysis included 20,755 (67.6%) after excluding names with an uncertain author gender. Multivariate analysis of the independent variables revealed that all variables: the author’s position, the publication year, 10 selected ophthalmology journals, except the IF (*p* = 0.76) had a significant effect (*p* < 0.001) on the proportion of women authors.

Women comprised 8,995 (43.3%) of the authors in all journals: 2,097 (45.7%) of first author positions, 5,377 (45.4%) middle, and 1,521 (35.2%) last (senior) authors. The percentage of women in first and middle positions was significantly higher than in the last position (*p* < 0.001).

The percentage of women authors in each author’s position significantly increased over time from 37.7 to 47.1% (*P* < 0.001). We observed a significant increase in the percentage of women authors as the journal’s impact factor (IF) increased (*P* < 0.001).

**Fig. 1:**
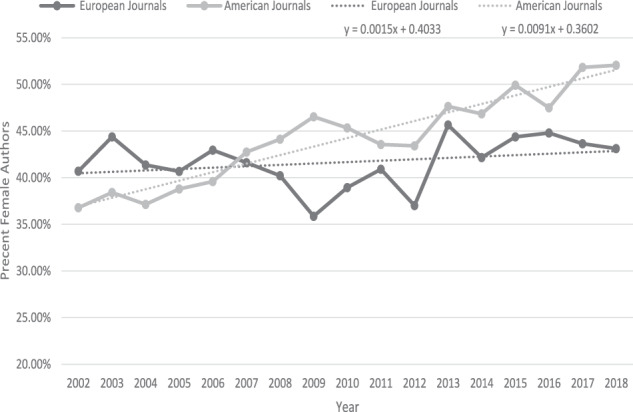
The distribution of 10 selected ophthalmology journals in comparison to 5 European journals and 5 American journals from 2002 to 2018. The gray solid and dotted lines represent the American journals. The black solid and dotted lines represent the European journals. Each dotted line constitute the linear regression of the solid line graph in the same colour.

There was a significant rise over time (*P* < 0.001) in the percentage of women authors in American and European journals, with a steeper slope for American journals and Eye (44.5%) than for the other European journals (41.9%) (Fig. 1).

We found a significant difference in the percentage of women authors between journals rated as Q1 or Q2 (42.2%), Q3 or Q4 (45.2%), and unrated journals (44.7%) (*P* = 0.002).

Our results indicate a rise in the number of women authors in the field of POS from 2002 to 2018. However, women are still underrepresented in the senior investigator position in comparison with their male counterparts. Furthermore, the increase was less evident in higher ranking journals. An encouraging finding, however, was the fact that female pediatric ophthalmologist authorship increased over time more than in general ophthalmology [1, 2]. In addition, despite an overall increase in the contribution of women to the field of POS, the increase was significant in all American journals; it was not significant in most leading European journals perhaps due to conservative cultural views of women’s roles in European society [3].

There is still a need to increase the participation of women in POS scientific publications, especially as leading authors, in better ranked publications, and in European scientific journals.

## References

[1] Franco-Cardenas V, Rosenberg J, Ramirez A, Lin J, Tsui I (2015). Decadelong profile of women in ophthalmic publications. JAMA Ophthalmol.

[2] Mimouni M, Zayit-Soudry S, Segal O, Barak Y, Nemet AY, Shulman S (2016). Trends in authorship of articles in major ophthalmology journals by gender, 2002–2014. Ophthalmology.

[3] Sieverding M, Eib C, Neubauer AB, Stahl T. Can lifestyle preferences help explain the persistent gender gap in academia? The “mothers work less” hypothesis supported for German but not for U.S. early career researchers. *PLOS ONE*. 2018:1–18. 10.1371/journal.pone.0202728. 10.1371/journal.pone.0202728 PMC6112653 30153285

